# Exosomal Circsafb2 Reshaping Tumor Environment to Promote Renal Cell Carcinoma Progression by Mediating M2 Macrophage Polarization

**DOI:** 10.3389/fonc.2022.808888

**Published:** 2022-05-12

**Authors:** Xin Huang, Jingyu Wang, Jibin Guan, Zhong Zheng, JunFeng Hao, Zitong Sheng, Menghua Wang, Tianhua Xu, Guangying Guo, Li Yao

**Affiliations:** ^1^ Department of Nephrology, The First Hospital of China Medical University, Shenyang, China; ^2^ Masonic cancer center, University of Minnesota, Minneapolis, MN, United States; ^3^ Department of Chemistry Justus Liebig University Giessen, Giessen, Germany; ^4^ Institute of Nephrology, Affiliated Hospital of Guangdong Medical University, Zhanjiang, China

**Keywords:** renal cell carcinoma, exosome, CircSAFB2, M2 macrophage, miR-620/JAK1/STAT3

## Abstract

**Background:**

Macrophages are the most abundant infiltrating immune-related stromal cells present in and around tumors, showing different phenotypes and functions. M2 macrophages mainly exert immunosuppressive functions and promote tumor growth. Exosomes are emerging as important mediators of cross-talk between tumor cells and the microenvironment. CircRNAs are novel members of non-coding RNAs that regulate cancer proliferation and progression. However, the mechanism by which exosomal circRNA regulates macrophage polarization in renal cell carcinoma (RCC) is still largely unknown.

**Methods:**

RCC-derived exosomes were characterized using transmission electron microscopy and nanoparticle tracking analysis (NTA). CCK-8, wound healing, and Transwell assays were performed to assess whether exosomes would affect the proliferation, migration, and invasion of RCC. Furthermore, we performed a bioinformatics analysis to identify circRNAs in RCC serum-derived exosomes from the GEO database. The fluorescence *in situ* hybridization (FISH) assay was used to detect the cellular distribution of circSAFB2. Bioinformatics analyses (StarBase 2.0) were used to pool the miRNA targets of circSAFB2. Luciferase assays were performed to verify the direct interactions. Western blotting was used to detect markers of macrophage M2 polarization. Lastly, mouse xenograft and bioluminescence imaging were used to examine the clinical relevance of exosomal circSAFB2 *in vivo*.

**Results:**

We report the circRNA derived from SAFB2 and evaluate its biological function in promoting the immune escape of RCC. We found that circSAFB2 was highly expressed in RCC tissues and RCC-derived exosomes. Furthermore, we demonstrated that exosomal circSAFB2 mediates the polarization of M2 macrophages through the miR-620/JAK1/STAT3 axis to promote RCC metastasis.

**Conclusions:**

Our data first demonstrated that circSAFB2 leads to immune escape from RCC by mediating M2 macrophage polarization *via* the miR-620/JAK1/STAT3 axis. These findings indicate a novel molecular mechanism of exosomal circSAFB2 in the progression of RCC and implicate circSAFB2 as a target for exosome-mediated tumor immune evasion.

## Introduction

Renal cell carcinoma (RCC) accounts for approximately 3% of all adult cancers, and the survival rate of patients with RCC is poor, especially in patients with metastases (1). The high mortality in patients with RCC is mainly due to the difficulty of early diagnosis, local invasion, and early metastasis (2). Therefore, it is essential to discover new diagnostic biomarkers and understand the molecular mechanisms underlying RCC metastasis.

Recently, the function of exosomes in cancer progression has attracted increasing attention. Exosomes are small bilayer lipid membrane vesicles with a diameter of approximately 30 to 100 nm secreted by various types of cells (3, 4). Increasing evidence shows that cancer-derived exosomes play a key role in the communication between tumor cells by transferring and exchanging carcinogenic molecules, including circRNA, microRNA (miRNA), mRNA, protein, and lipid, and thus participate in the promotion of tumorigenesis and tumor proliferation, tumor metastasis, angiogenesis, immune escape, and drug resistance (5, 6). Circular RNAs (circRNAs) have been identified as members of the non-coding RNA family (ncRNAs), which have a closed-loop structure and have no 5’ or 3’ ends (7). In addition, circRNAs are enriched in exosomes and have been found to be important for cell-to-cell communication. Studies have found that exosomal circRNAs are stable in the blood, which may be a promising new biomarker for clinical diagnosis (8). CircRNAs have been reported to be effective miRNA sponges, regulating the expression of downstream target genes by competitively inhibiting miRNAs (9, 10). Exosomal circRNAs has been recognized as a new class of potential biomarkers and therapeutic targets. However, the functions and underlying molecular mechanisms of cancer-derived exosomal circRNAs remain largely unexplored.

Macrophages are the most abundant immune-related infiltrating stromal cells present in and around tumors, showing different phenotypes and functions (11). Macrophages can be polarized into classically activated cells (M1) or alternatively (M2) depending on environmental signals. M1 macrophages are defined by their pro-inflammatory phenotype, expressing numerous pro-inflammatory mediators, including IL-1β, IL-1α, IL-12, tumor necrosis factor (TNF)-α, which tends to inhibit tumor development. In contrast, M2 macrophages produce type II cytokines, such as IL-4, IL-6, and IL-10, which mainly exert immunosuppressive functions and promote tumor growth (12). However, the mechanisms underlying macrophage polarization remain largely unknown in RCC. In this study, we found that circSAFB2 is highly expressed in RCC, which is the first exploration of the function of circSAFB2, CircSAFB2 was generated by back-splicing of the exons 10 and 11 of the SAFB2 gene with several Alu elements within the introns on both sides. We demonstrate that exosomal circSAFB2 derived from RCC can polarize macrophages *via* the miR-620/JAK1/STAT3 signaling pathway. Polarization of M2 macrophages leads to an increased metastatic potential of RCC *in vitro* and *in vivo*. Therefore, the data implicate circSAFB2 as a target for exosome-mediated tumor immune evasion.

## Materials and Methods

### Patient Samples and Ethical Statement

Forty paired RCC tumors and corresponding adjacent non-cancerous tissues and 60 RCC blood samples were collected from the First Affiliated Hospital of China Medical University. For each tumor, the age, sex, tumor size (cm), TNM stage, and lymph node status are shown in **Table 1**. The study was approved by the Ethics Committee of the First Affiliated Hospital of China Medical University.

**Table 1:** Relationship between circSAFB2 expression and clinicopathological parameters of patients with RCC.

Variable	No. of patients	CircSAFB2 expression		*P*-value
		Low (n=30)	High (n=30)	
Gender				
Female	29	17	12	0.196
Male	31	13	18	
Age (year)				
≤60	28	12	16	0.301
>60	32	18	14	
Tumor size (cm)				
≤5	26	17	9	0.037
>5	34	13	21	
TNM stage				
I+II	33	21	12	0.020
III+IV	27	9	18	
Lymph node metastasis				
Negative	24	15	9	0.045
Positive	36	13	23	

### Cell Culture

Six human RCC cell lines (A498, 786-O, Caki1, Caki2, 769-P, and ACHN) were cultured in DMEM medium (Biological Industries, Shanghai, China) supplemented with 10% fetal bovine serum (FBS, Biological Industries, USA), penicillin (100 U/mL), streptomycin (100 g/mL). These RCC cell lines were cultured at 37°C in an incubator with a humidified atmosphere containing 5% CO2.

### Human THP-1 Cell Culture

The human acute monocytic leukemia cell line THP-1 was cultured in RPMI 1640 medium supplemented with 0.05 mM 2-mercaptoethanol and 10% heat-inactivated FBS at 37°C in an incubator with 5% CO2. Macrophages were collected after 72 hours of the culture of THP-1 in RPMI 1640 medium (Gibco by Life Technologies, Grand Island, NY, USA) and treated with PMA (80 nM).

### Plasmid Generation and Cell Transfection

The siRNAs targeting circSAFB2, JAK1, STAT3 and miR-620 mimics or inhibitors were all designed and synthesized by GeneChem (Shanghai, China). The cells were then transfected using the Lipofectamine™ 3000 (ThermoFisher Scientific, USA) reagent according to the manufacturer’s instructions.

### Isolation of Exosomes

Exosome isolation was performed by differential ultracentrifugation as previously described. Briefly, 50 mL of cell cultures were centrifuged at 4°C to obtain the supernatant, which was subjected to centrifuge at 10,000×g for 20 min. The resulting supernatant was subsequently transferred to a sterile centrifuge tube. Thereafter they were centrifuged at 100,000×g at 4°C for 70 min. The supernatant was collected and the sediments were resuspended with 1x PBS and filtered with a 0.22-μm strainer. The liquid obtained was then centrifuged at 100,000×g for 1 hour. The previous step was repeated until the exosomes could be collected. Plasma exosomes were isolated from 500 μL of fresh plasma collected from each RCC patient as described above.

### Transmission Electron Microscopy

The isolated exosomes suspended in 100 μL of 1× PBS were fixed with 4% paraformaldehyde. The exosomes were then dropped onto Formvar carbon-coated 400 mesh copper electron microscopy grids and fixed with 1% glutaraldehyde for 20 min. Thereafter, the samples were negatively stained with a 2% uranyl-oxalate (pH 7) for 5 min and subsequently with a 9:1 ratio of 2% methylcellulose (pH 4) and 4% uranyl acetate for another 10 min. After air-drying of the grids, micrographs were captured under the FEI TecnaiG2 spirit transmission electron microscope (ThermoFisher, Waltham, MA, USA) operated at 80 kV.

### Nanoparticle Tracking Analysis

Dynamic light scattering was performed using a zeta potential and particle size analyzer (elsz-2000; Otsuka Electronics Co., Ltd., Osaka, Japan). The exosomes were resuspended in PBS. The size, distribution and quantity of particles in the exosome preparation were were analyzed with a 301 NanoSight NS300 system (Malvern Instruments, Malvern, UK) as previously described. All experiments were conducted at a dilution of 1:1000, providing a particle concentration of about 6 ×10^7^/mL. Each experiment was conducted in triplicate.

### Exosome Labeling and Tracking

Purified exosomes isolated from the culture medium were collected and labeled with PKH26 red fluorescent membrane linker dye (Sigma, USA) according to the manufacturer’s instructions. The labeled exosome pellets were then resuspended and added to unstained macrophage cells for exosome uptake studies. They were incubated for 30 minutes, 2 hours or 12 hours at 37°C. After this, the cells were washed twice with PBS and fixed. The nuclei were stained with 4 ‘, 6-diamidino-2-phenylindole (DAPI). Finally, the cell samples were measured by fluorescence microscope.

### Transwell Assay

The Transwell invasion and migration assay was performed in 24-well plates (Corning, MA, USA), using a 6.5-mm diameter Transwell chamber with 8-mm Transwell inserts (Corning). Tumor cells (5×10^4^) were suspended in 200 mL of serum-free medium and seeded in the bottom of the upper chambers for migration assays. A medium containing 10% FBS was added to the bottom chamber. For the invasion assay, the insert membranes were coated with Matrigel (50 mL/well) (BD Biosciences, USA) before adding the cells. After 24-hour culture, cells were stained with 0.1% crystal violet for 30 minutes, and nonmigrating or non-invading cells were removed. Six visual fields were randomly selected for counting the number of migrated cells.

### Wound Healing Assay

RCC cells were treated with or without exosomes for 24 hours; RCC cells were treated with or without exosome-treated macrophage cell-conditioned medium for 24 hours; RCC cells were then harvested and seeded onto a 6-well plate (2×10^5^ cells per well). A single scratch wound was generated using a sterile 200-μL pipette tip, and floating cells were removed by washing with 1×PBS. The scratches were observed and photographed using an inverted microscope Nikon Inverted Research Microscope Eclipse Ti microscope at 100× magnification at 0 and 24 hours after scratching.

### Cell Counting Kit-8 Assay

RCC cells were treated with or without exosome-treated macrophage cell-conditioned medium for 24 hours; RCC cells (2×10^3^) were seeded in 96-well plates (Corning). Next, 10 μL of Cell Counting Kit-8 assay (CCK-8) solution (Beyotime, Jiangsu, China) was added to each well at the appointed time. After incubation for one hour at 37°C, absorbance at 450 nm was measured using an automatic microplate reader (Synergy4; BioTek, Winooski, VT, USA).

### Western Blotting

Cell proteins were extracted with RIPA lysis buffer (Thermo Fisher, USA). The protein was extracted by incubation, vortex, and centrifugation (15,000 ×g, 4°C for 25 min). A BCA reagent kit (Beyotime, China) was used to measure the protein concentrations. Total protein was separated by SDS-PAGE gel (100 V, 1.5 h) and transferred onto polyvinylidene difluoride (PVDF) membranes (Millipore, USA) (50 V, 80 min). After blocking in 5% nonfat milk for 1 hour, the membranes were incubated overnight at 4°C with the indicated primary antibodies, including anti-CD63 (Abcam, ab68418), anti-TSG101 (Abcam, ab133586), anti-E-cadherin (Abcam, ab76055), anti-N-cadherin (Abcam, ab76011), anti-vimentin (Abcam, ab20346), anti-JAK1 (Abcam, ab138005), anti-STAT3 (Abcam, ab68153), IL-10 (Abcam, ab215975), arginase-1 (ab133543) and anti-GAPDH (Abcam, ab9485). They were then incubated with secondary antibodies for 1 hour at room temperature and visualized by the ECL chemiluminescence reagent (Millipore, USA).

### Bioinformatics Analysis

Differentially expressed circRNA were screened from the GEO database (GSE100206 and GSE100207). CircSAFB2 sequence data were obtained from circBase (http://www.circbase.org/). The target miRNAs of circSAFB2 and the target gene of miRNAs were predicted using starBase 2.0 (http://starbase.sysu.edu.cn/index.php).

### Quantitative Reverse Transcription-Polymerase Chain Reaction

Total RNA was extracted from the cells or tissues using the Trizol and enzyme RNA Extraction Kit (Takara, RR820A, China). RNA (1 μg) was reverse-transcribed using a reverse transcription Kit (Takara, RR047A, China). Subsequently, quantitative real-time PCR (qRT-PCR) was performed with gene-specific primers (Biotechnology) on the 7500ABI biological system machine. The comparison threshold was used to calculate the absolute mRNA number. U6 (Rnu6-1) small nuclear RNA was used as an endogenous control for miRNA and GAPDH was used as an internal control for mRNA, and the results of each sample were normalized to the expression of either U6 or GAPDH.

### Luciferase Reporter Assays

CircSAFB2wt/mut or JAK1wt/mut were co-transfected with miR-620 mimic or control in M cells with Lipofectamine TM 3000 (Invitrogen, CA, USA). in the ratio of 15:1, and stranded at room temperature for 5min. Cell lysates were collected and analyzed 48 hours after transfection. The activities of the firefly and Renilla luciferase were measured using the Dual-Luciferase reporter gene detection kit (Promega, USA) according to the manufacturer’s protocol. Luciferase activity was measured by a double luciferase reporter gene detection system. All luciferase values were standardized with those of Haishen luciferase and expressed as multiple changes relative to basic activity. The OD value was observed by the enzyme labeling instrument.

### Immunohistochemistry

Immunohistochemistry (IHC) staining was performed using the streptavidin-biotin-peroxidase complex method. In short, renal cancer tissue samples were fixed in 4% paraformaldehyde for 3 days and dehydrated with gradient alcohol. Then, it was immersed in an ethanol-xylene (1:1) mixture for 15–20 min, followed by xylene until transparent. The transparent tissue was immersed in a mixture of paraffin and xylene (1:1) for 1 h, followed by paraffin-embedding. The paraffin sections were sliced into 5-μm-thick sections and dried in a 37°C incubator, following the routine dehydration, transparency, and mounting. Renal tissue paraffin slice (4 μm) was washed, followed by antigen thermal retrieval, incubation with primary antibodies, CD63 antibody (Proteintech, 25682-1-AP) at 4°C overnight. It was then incubated in a secondary biotinylated antibody for 30 minutes at 37°C, and finally, visualized with DAB solution and counterstained with hematoxylin. Finally, the paraffin sections were covered with coverslips for microscopic observation (Nikon, Japan) observation.

### RNA Fluorescence *in Situ* Hybridization

The Cy3-labeled circSAFB2 probes used in the present study were designed and synthesized by RiboBio (Guangzhou, China). The signals were detected by the FISH Kit (RiboBio) according to the manufacturer’s instructions. The macrophages were first permeabilized in PBS with 0.5% Triton X-100. The cells were then hybridized in a hybridization buffer solution with specific fluorescence *in situ* hybridization (FISH) probes at 37°C overnight. The hybridization buffer was then gradually washed with 4× SSC (including 0.1% Tween-20), 2× SSC and 1× SSC at 42°C. Then the nucleus was stained with DAPI for 10 minutes. Finally, the results were observed with a TCS SP2 AOBS confocal laser microscope (Leica Microsystems, Germany).

### RNA Pull-Down

The biotin-labeled circSAFB2 probe and the negative control probe were synthesized by RiboBio Biotech (Guangzhou, China), and the sequences are 5’-GGAAGAGCCGTGAGGCCGAGCCAGTAGTTCGGTGATTGTAGATATGAAGTT-3’. Macrophages cells were transfected with a vector or control vector overexpressing circSAFB2. After 48 hours, the biotinylated-circ SAFB2 probe was incubated with C-1 magnetic beads (Life Technologies, Carlsbad, CA, USA), generating probe-coated beads. The coated beads were then incubated with sonicated Mcells at 4°C overnight. The bound RNA complex is eluted from the beads and purified using the RNeasy Mini Kit (Qiagen, Valencia, CA, USA). The abundance of miR-620 was detected by qRT-PCR analysis.

### Flow Cytometry

We collected peripheral blood from healthy volunteers, separated peripheral blood mononuclear cells (PBMCs) by Ficoll density gradient centrifugation, and sorted CD14^+^ monocytes from PBMCs using CD14 microbeads (Miltenyi 130-050-201), and used 100 ng/ml M-CSF (HY-P7050, MedChemExpress, China) induces monocytes into macrophages. RCC exosomes and exosomes overexpressing circSAFB2 were co-cultured with macrophages, and after 24 hours, flow cytometry was used to analyze the expression level of M2 macrophages, marked by CD206.

### *In Vivo* Metastasis

Male nude mice (6 weeks old) were maintained for the metastasis experiments according to standard guidelines. All animal experiments were approved by the Ethics Committee of the First Affiliated Hospital of China Medical University. To study the role of RCC-derived exosomes in RCC metastasis, mice received an injection into the tail vein containing control and exosomes treated-ACHN luciferase cells (1×10^6^) (n=6/per group). To study the role of exosome-induced Treg expansion derived from RCC in RCC metastasis, we mixed ACHN luciferase cells (1×10^6^) (n=6/group) with conditioned macrophage cells stimulated by exosomes with indicated treatment and injected them into the mouse *via* tail vein to observe distant metastasis. Bioluminescence was monitored and recorded every week. After about 4 weeks, D-luciferin (The *In vivo* Imaging Community) at a dose of 1.5 mg/10 g body weight was injected intraperitoneally into mice, and 10 min later, representative bioluminescence imaging of metastases was measured with the IVIS imaging system (Xenogen IVIS 2000 Luminal Imager, Xenogen Corp., Alameda, CA, USA). We used living image ver. 2.6 (Xenogen) software acquisition and analysis of data.

### Statistical Analysis

SPSS 25.0 software (SPSS Inc., USA) was used for statistical analysis. All data were presented as the mean ± standard deviation (SD) of at least three independent experiments. The graphs were generated in GraphPad Prism 8.0 (GraphPad Software, USA). Student’s t-test and one-way ANOVA were used to determine the statistical significance for comparisons of 2 or more groups. Differences were considered statistically significant at a *P*-value <0.05.

## Results

### RCC-Derived Exosomes Enhance RCC Proliferation, Migration, and Invasion

**Figure 1:**
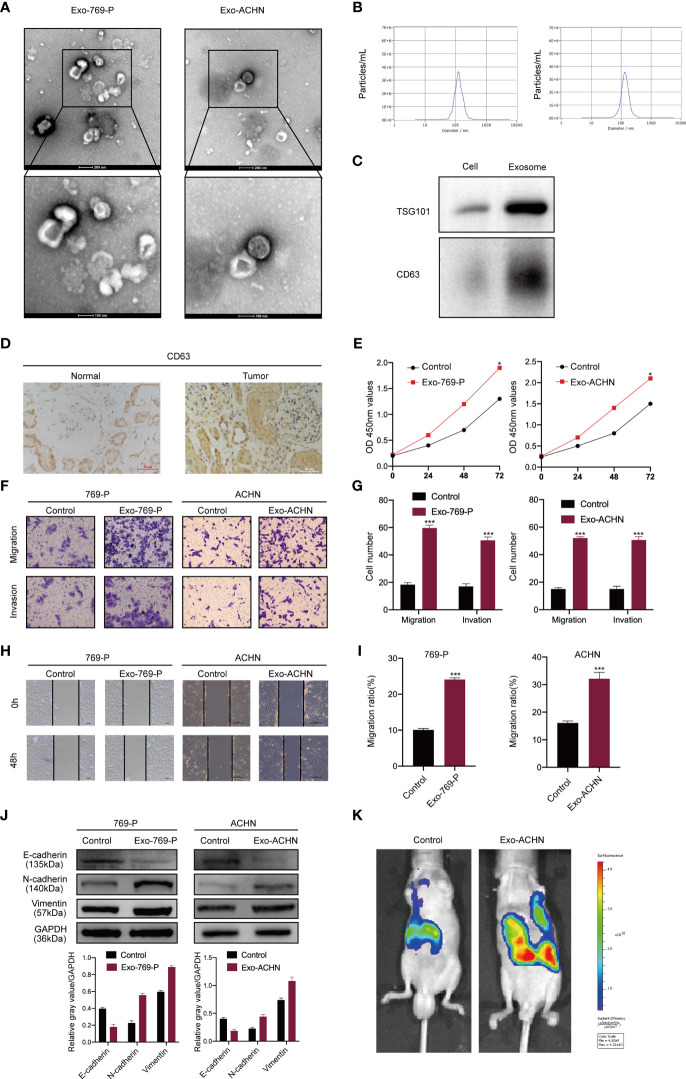
RCC-derived exosomes enhance RCC proliferation, migration, and invasion. **(A)** Exosomes isolated from the supernatant of the culture medium of 769-P and ACHN cells. The morphology and the average size were confirmed by transmission electron microscopy (scale bar=500 nm and 100 nm); **(B)** NTA distribution of RCC cell-derived exosomes; **(C)** Western blotting of exosome markers (CD63, TSG101); **(D)** The expression of CD63 was examined by IHC in normal and RCC tissues; **(E)**. The proliferation capacity of 769-P and ACHN cells treated with or without RCC cell-derived exosomes was determined by the CCK8 assay at 0, 24, 48, and 72 hours; **(F)** Migration and invasion capacity of RCC cells (769-P and ACHN) treated with or without exosomes using Transwell; **(G)** Morphometric analysis of migratory and invaded cells; **(H)** Scratch wound healing assays in 769-P and ACHN cells treated with or without exosomes; **(I)**. Statistical analysis of cell migration in scratch wound healing assays; **(J)** Western blotting and gray value of EMT-related protein expression in 769-P and ACHN cells after exosome stimulation. GAPDH as the loading control; **(K)** Images of nude mice were injected through the tail vein with control and exosome-treated exosomes treated-ACHN cells (n=6/per group). Metastasis was monitored and imaged by bioluminescence using an *in vivo* imaging system. All data were reported as the mean ± SD; *P < 0.05, ***P < 0.001.

Previous studies have revealed that RCC-derived exosomes are associated with tumor proliferation and metastasis (13). To verify the mechanism of exosomes in RCC, we isolated cancer cell-derived exosomes from the supernatant of two RCC cell lines, 769-P and ACHN. Then, transmission electron microscopy and NTA were used to identify these exosomes. The results showed that exosomes were round particles with a size of about 80–100 nm and a double-layer membrane structure, which was consistent with common sizes of exosomes previously reported (**Figures 1A, B**). We also performed western blot identification of exosome markers (**Figure 1C**). To explore whether exosomes influence the proliferation, migration, and invasion of RCC cells. We first detected the expression of CD63 (exosomes marker) by IHC in tissues from renal cancer. CD63 expression in tumor tissues was significantly higher than in nontumor liver tissues (**Figure 1D**). The CCK8 assays showed that the exosomes significantly improved the proliferation of RCC cells compared to the control groups (**Figure 1E**). Furthermore, wound healing and transwell analysis also showed that compared to the control group, RCC cell exosomes could significantly promote the migration and invasion of RCC cells (**Figures 1F–I**). Furthermore, western blotting results showed that RCC-derived exosomes had decreased epithelial cell marker expression (E-cadherin) and increased mesenchymal cell marker expression (N-cadherin, vimentin) (**Figure 1J**). We also tested the influence of exosomes on metastases using an *in vivo* metastasis model. Control cells and exosomes treated with ACHN-luciferase were injected into the tail vein of nude mice. Then, a live animal bioluminescence imaging system was used to monitor tumor metastasis weekly. We found that exosome treatment significantly increased ACHN cell metastasis (**Figure 1K**). These results indicated that RCC-derived exosomes promoted RCC proliferation, migration, and invasion.

### RCC-Derived Exosomes Enhance RCC Proliferation, Migration, and Invasion by Inducing Macrophage M2 Polarization

**Figure 2:**
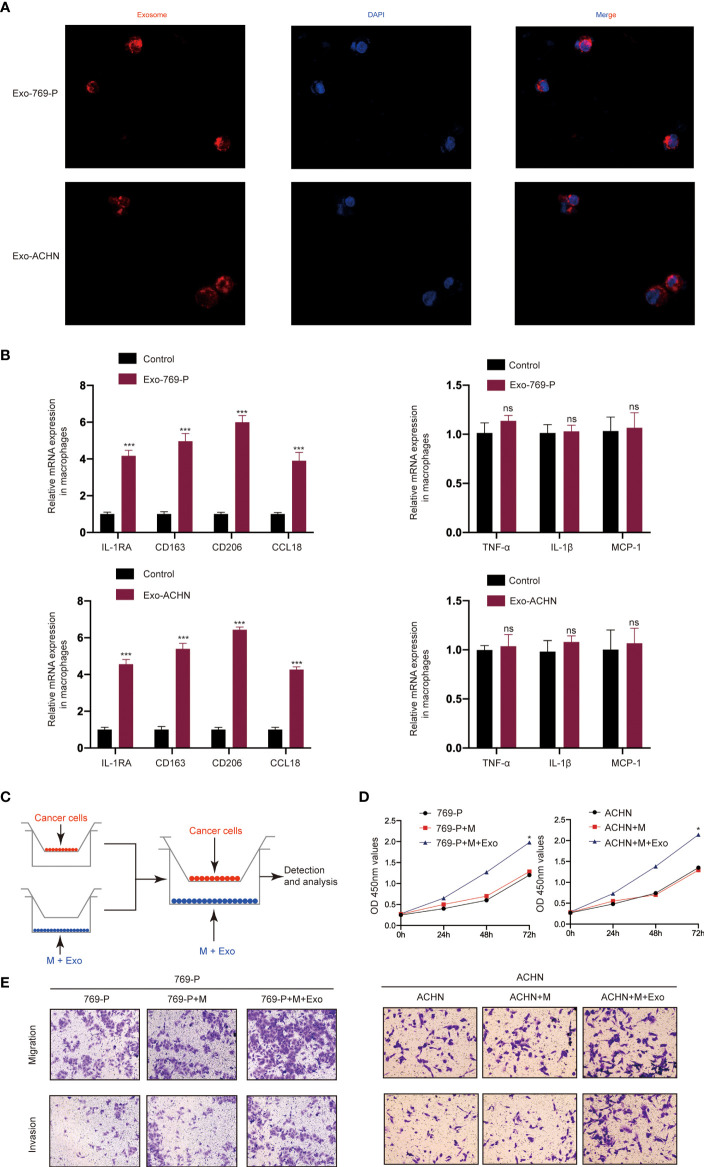
RCC-derived exosomes enhance RCC proliferation, migration, and invasion by inducing M2 macrophage polarization: **(A)** Representative immunofluorescence image shows the internalization of PKH26-labeled RCC (769-P and ACHN)-derived exosomes (red) by macrophages; **(B)** qRT-PCR of the expression of M2-like macrophage markers (IL-1RA, CD163, CD206 and CCL18) and M1-like macrophage markers (TNF-α, IL-1β and MCP-1); **(C)** Schematic illustration of the indirect *in vitro* co-culture system; **(D)** Cell proliferation capacity of RCC cells (769-P and ACHN) co-cultured with macrophages treated with exosomes was determined by CCK-8; **(E)** Migration and invasion capacity of RCC cells (769-P and ACHN) co-cultured with macrophages treated with or without exosomes using Transwell; All data were reported as the mean ± SD; *P < 0.05, ***P < 0.001. ns, no significance..

We then investigated whether RCC-derived exosomes could induce macrophage polarization. The isolated exosomes from 769-P and ACHN cells were labeled with PKH26 dye and incubated with macrophages (THP-1+PMA) for 48 hours. A confocal microscope was then used to track the movement of exosomes. The results show that there was a strong red signal in the receiving cells, indicating that the exosomes were taken up by the recipient cells (**Figure 2A**). Furthermore, qRT-PCR results showed that RCC-derived exosomes increased the mRNA expression levels of markers of M2-like macrophages, including IL-1RA, CD163, CD206 and CCL18 (**Figure 2B**), while there was no significant effect on the mRNA expression levels of markers of M1-like macrophages, including TNF-α, IL-1β and MCP-1. Next, we studied the role of M2 polarization of macrophages induced by RCC-derived exosomes in the proliferation, migration, invasion of RCC using an *in vitro* indirect co-culture system (**Figure 2C**). The CCK8 assays showed that exosome-treated macrophages significantly increased RCC cell proliferation (**Figure 2D**). Furthermore, transwell analysis showed that macrophages treated with exosomes could significantly promote migration and invasion of RCC cells (**Figure 2E**). Collectively, our data demonstrated that activated M2 macrophages induced by RCC-derived exosomes promote the proliferation, migration, and invasion of RCC.

### CircSAFB2 Is Significantly Up-Regulated in Macrophages Following the Addition of Tumor-Derived Exosomes

**Figure 3:**
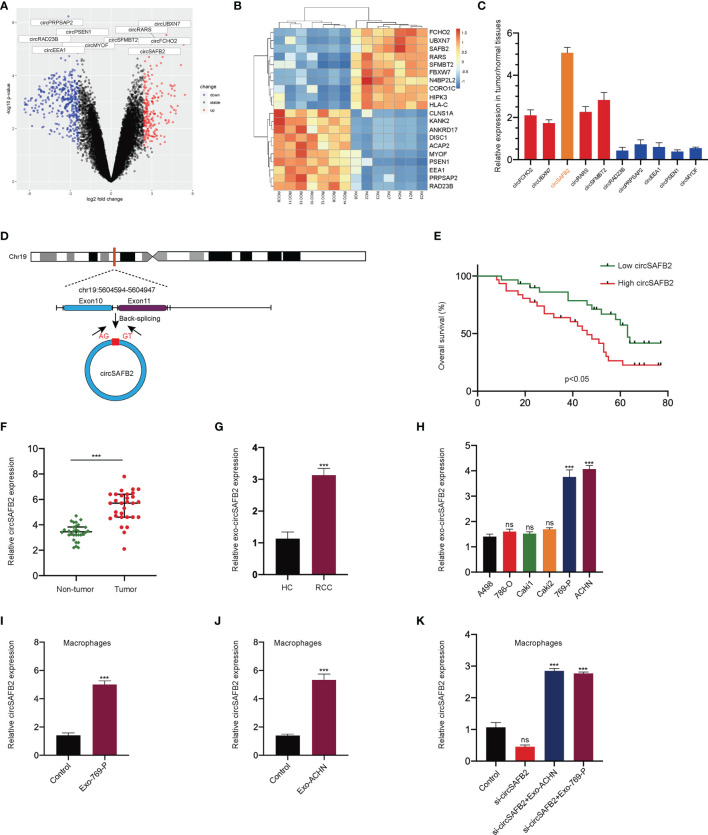
circSAFB2 is significantly up-regulated in macrophages with the addition of tumor-derived exosomes. **(A)** Volcano map of the circRNA expression profile composed of 7 pairs of RCC patients and healthy individuals; **(B)** Heat map of the circRNA expression profile composed of 7 pairs of RCC patients and healthy individuals; **(C)** The expression of the top 5 up-regulated and down-regulated circRNAs in 10 paired RCC tumors and normal tissues by qRT-PCR; **(D)**. Structure of the elements circSAFB2 in the flanking sequence; **(E)** Kaplan–Meier analysis of overall survival in 60 patients with RCC according to circSAFB2 expression (log-rank test); **(F)** Expression of circSAFB2 in 30 paired RCC tissues and adjacent non-tumor tissues; **(G)** Expression of circSAFB2 in 30 RCC patients and 30 healthy individuals; **(H)** Circulation level of circHIPK3 expression in six renal cancer cell lines (A498, 786-O, Caki1, Caki2, 769-P, and ACHN); **(I, J)**. qRT-PCR analyses of circSAFB2 in macrophages after RCC-derived exosomes (769-P and ACHN cells); **(K)** qRT-PCR analyses of circSAFB2 in macrophages after transfection with si-NC or si-circSAFB2 with or without the addition of RCC-derived exosomes (769-P and ACHN cells) addition. All data are reported as mean ± SD; n=3, ***P < 0.001. ns, no significance.

The GSE108735 microarray was applied to explore several differentially expressed circRNAs between RCC and normal tissues. Variation in circRNA expression was demonstrated in the volcano plot on the basis of log_2_ (fold changes) ≥1, *p*<0.05 (**Figure 3A**). The top 10 up-regulated and down-regulated circRNAs are shown on the heat map (**Figure 3B**). Then, we further analyzed the expression of the top 5 up-regulated and down-regulated circRNAs in 10 paired RCC tumors and normal tissues by qRT-PCR. The qRT-PCR results showed that circSAFB2 was the most differentially expressed circRNA in RCC (**Figure 3C**). CircSAFB2 was generated by back-splicing of the exons 10 and 11 of the SAFB2 gene with several Alu elements within the introns on both sides (**Figure 3D**). Next, Kaplan–Meier survival analysis indicated that patients with high circSAFB2 expression showed significantly shorter average survival times than patients with low expression of circSAFB2. CircSAFB2 expression in RCC tissues was significantly higher than that of adjacent non-tumor tissues (**Figure 3E**). Furthermore, we examined the expression of circSAFB2 by qRT-PCR in 30 paired RCC tissues and adjacent non-tumor tissues (**Figure 3F**). Then we detected the difference in the expression level of circSAFB2 in blood exosomes between RCC and healthy control. qRT-PCR results showed that the expression of circSAFB2 in exosomes derived from RCC was significantly higher than that of the healthy control (**Figure 3G**).

Then, qRT-PCR analysis in exosomes derived from six RCC cell lines showed that circSAFB2 was significantly up-regulated in ACHN and 769-P cell lines compared to other cell lines (**Figure 3H**). In addition, we explored the effects of exosomal circSAFB2 derived from ACHN and 769-P cell lines on circSAFB2 in macrophages. The results of qRT-PCR showed that after the exosomes derived from ACHN and 769-P cell lines were co-cultured with macrophages, circSAFB2 in macrophages was significantly higher than in the control group (**Figures 3I, J**). To further verify that RCC-derived exosomes could carry circSAFB2 into macrophages, we transfected circSAFB2 siRNA into macrophages. qRT-PCR assays validated that knockdown of circHIPK3 resulted in a significant decrease in its expression. After co-treatment with ACHN and 769-P-derived exosomes, circSAFB2 expression increased significantly in macrophages (**Figure 3K**). These results suggest that circSAFB2 is significantly up-regulated in macrophages with the addition of tumor-derived exosomes.

### CircSAFB2 Serves as a Sponge for MiR-620

**Figure 4:**
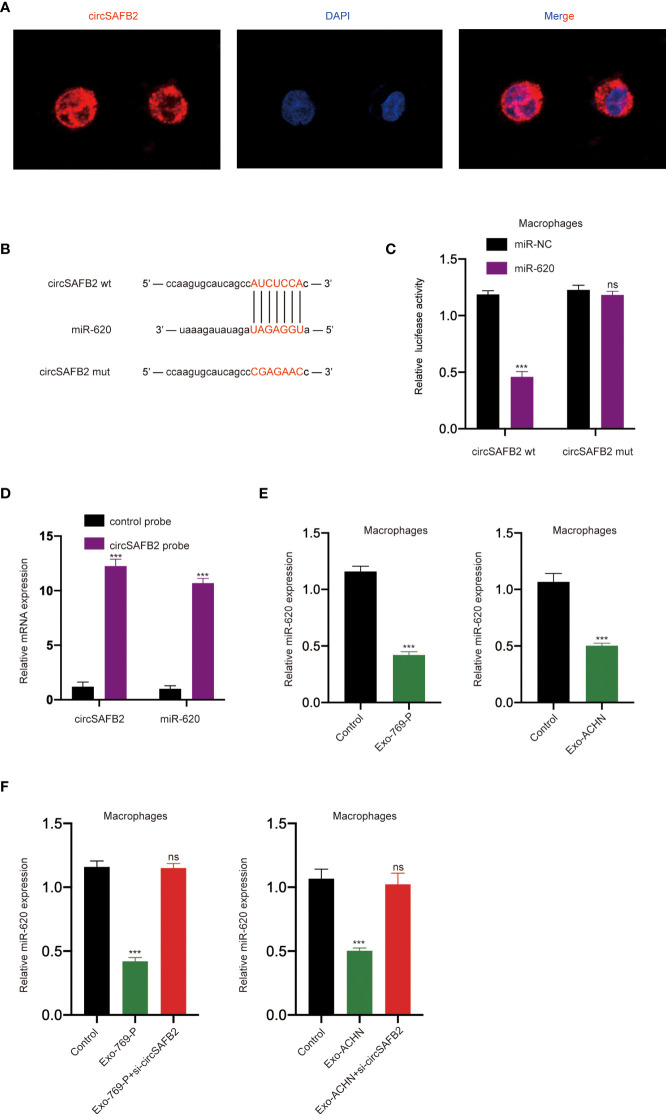
circSAFB2 serves as a sponge for miR-620. **(A)** Localization of circSAFBin macrophages after treatment with exosomes detected by FISH; **(B)** Schematic representation of the 3′-UTR of circSAFB2 with the predicted target site for miR-620. The mutant site of circSAFB2 3′-UTR is indicated (without line); **(C)** A luciferase reporter analysis was performed to examine the binding ability between circSAFB2 and miR-620. Reporter constructs containing circSAFB2wt or circSAFB2mut at the predicted miR-620 target sequences were co-transfected into macrophages, along with miR-620 mimics or miR-NC; **(D)** Lysates prepared from macrophages overexpressing circSAFB2 were incubated with biotinylated probes against circSAFB2 before performing an RNA pull-down assay. qRT-PCR was used to determine the level of circSAFB2 and miR-620; **(E)** qRT-PCR analyses of miR-620 expression in macrophages after exosome treatment (Exo-769-P and Exo-ACHN); **(F)** qRT-PCR analysis of miR-620 expression in macrophages treated with exosomes and exosomes knocked down circSAFB2. All data are reported as mean ± SD; n=3, ***P < 0.001. ns, no significance.

An increasing number of previous studies have shown that circRNA exists mainly in the cytoplasm and functions as a miRNA sponge (14). The FISH results show that the circSAFB2 transcription signal was mainly distributed in the cytoplasm of macrophages, while there is little hybridization signal in the nucleus (**Figure 4A**). To study the potential miRNAs related to circSAFB2, we used the StarBase 2.0 online bioinformatics database (http://starbase.sysu.edu.cn/) to predict the most potential complementary miRNAs. Among these predicted target miRNAs, circSAFB2 was considered to have binding sites for miR-620 (**Figure 4B**). The dual-luciferase reporter assay further confirmed that miR-620 acted as a target for circSAFB2 (**Figure 4C**). Furthermore, we conducted an RNA pull-down assay to evaluate whether circSAFB2 could directly capture miR-620. The biotinylated circSAFB2 probe significantly pulled down circSAFB2 and miR-620 in macrophages after overexpression of circSAFB2 (**Figure 4D**). Furthermore, we found that miR-620 expression was significantly reduced in macrophages after co-culture with RCC cell-derived exosomes compared to controls (**Figure 4E**). Additionally, we transfected circSAFB2 siRNA into ACHN and 769-P cell lines. The exosomes derived from RCC cells were then co-cultured with macrophages and the qRT-PCR results showed that the exosomes with circSAFB2 knockdown did not increase miR-620 expression in macrophages (**Figure 4F**).

### RCC-Derived Exosomal CircSAFB2 Induces Macrophage M2 Polarization Through the MiR-620/JAK1/STAT3 Axis

**Figure 5:**
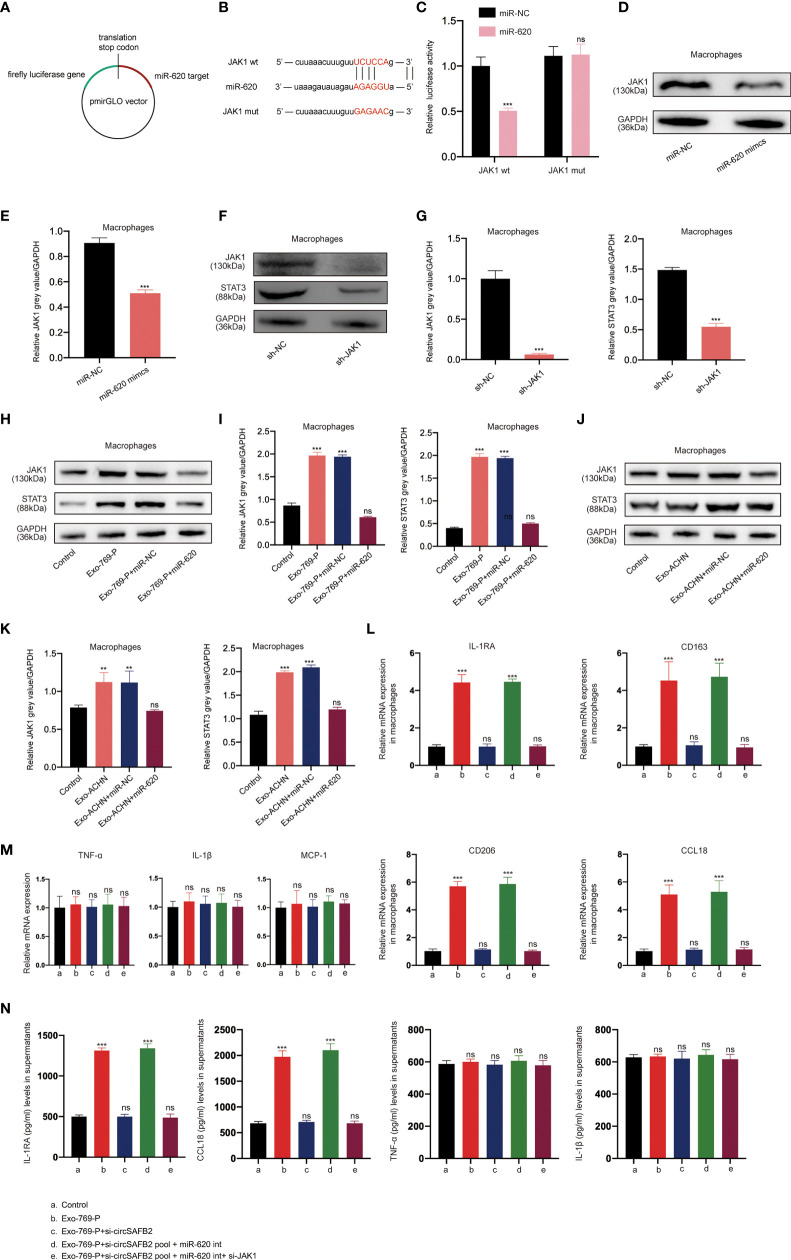
RCC-derived exosomal circSAFB2 induces M2 macrophage polarization through the miR-620/JAK1/STAT3 axis. **(A, B)** Schematic representation of the 3′- ‘UTR’ of JAK1 with the predicted target site for miR-620. The mutant site of JAK1 3′-UTR is indicated (without line); **(C)** Luciferase reporter analysis was performed to examine the binging capacity between miR-620 and JAK1. Reporter constructs containing JAK1wt or JAK1mut at the predicted miR-620 target sequences were co-transfected into macrophages, along with miR-620 mimics or miR-NC; **(D, E)** Western blotting and densitometric analysis of JAK1 expression in macrophages after transfection with miR-NC or miR-620 mimics; **(F, G)** Western blotting and densitometric analysis of JAK1 and STAT3 expression in macrophages after transfection with JAK1 siRNA; **(H–K)**. **(H, I)** Western blotting and densitometric analysis value of JAK1 and STAT3 expression in macrophages after treatment with 769-P-derived exosomes (Exo-769-P), along with miR-NC or miR-620 mimics. **(J, K)** Western blotting and densitometric analysis value of JAK1 and STAT3 expression in macrophages after treatment with ACHN-derived exosomes (Exo-ACHN), along with miR-NC or miR-620 mimics; **(L)**. qRT-PCR analysis of M2 macrophages (IL-1RA, CD163, CD206 and CCL18) after indicated treatment (Exo-769-P, Exo-769-P + si-circSAFB2, Exo-769-P + si-circSAFB2 pool + miR-620 int, Exo-769-P + si-circSAFB2 pool + miR-620 int + si-JAK1); **(M)**. qRT-PCR analysis of M1 macrophages (TNF-α, IL-1β and MCP-1) after indicated treatment; **(N)**. ELISA analysis of cytokine released from M2 macrophages (IL-1RA and CCL18) and M1 macrophages (TNF-α and IL-1β). All data indicate mean ± SD; n=3, **P<0.01, ***P<0.001. ns, no significance.

Studies have shown that miRNA can regulate the expression of mRNA and protein by binding to the 3’UTR of the target gene (15). The online bioinformatics tool StarBase 2.0 (http://starbase.sysu.edu.cn/) was used to predict the target genes of miR-620 (**Figures 5A, B**). We found that JAK1 was one of the best candidates. JAK1 has been reported to be associated with M2 polarization of macrophages (16). To evaluate the effect of miR-620 on JAK1 expression, we performed luciferase reporter assays to confirm that JAK1 is a target gene of miR-620. The results of the dual-luciferase reporter assay showed that miR-620 mimics significantly reduced the wild-type luciferase reporter activity of JAK1 (JAK1-wt) rather than the mutation luciferase reporter activity of JAK1 (JAK1-mt) (**Figure 5C**). The Western Blotting analysis also showed that miR-620 mimics significantly reduced JAK1 protein levels compared to those in the miR-NC group (**Figures 5D, E**). Increasing studies have shown that STAT3 is a classic downstream transcriptional activator of JAK1 (17). Therefore, we detected the protein expression level of STAT3 after JAK1 knockdown by western blotting, which indicated that the knockdown of JAK1 significantly reduced the expression of STAT3 (**Figures 5F, G**). To further confirm the regulatory effects of miR-620 on the JAK1/STAT3 signaling pathway, we treated macrophages with RCC-derived exosomes. We observed that JAK1 and STAT3 protein expression increased markedly in macrophages supplemented with RCC-derived exosomes, while up-regulation was significantly inhibited after co-treatment with the miR-620 inhibitor (**Figures 5H–K**). Furthermore, we explored whether RCC-derived exosomes could induce macrophages differentiation through miR-620. The qRT-PCR results found that RCC-derived exosomes significantly increased M2 macrophages markers expression (IL-1RA, CD163, CD206 and CCL18). However, exosomes that knocked down circSAFB2 could not increase M2 macrophages markers expression. The expression of M2 macrophage markers increased after co-culture with circSAFB2 knockdown exosomes and macrophages with miR-620 inhibitor. However, after further transfection of si-JAK1 in macrophages, the expression of M2 macrophages markers was reduced (**Figures 5L, M**). Additionally, none of the treatments had a significant effect on the expression of M1 macrophage markers (TNF-α, IL-1β and MCP-1) (**Figure 5N**). In addition, the results of flow cytometry analysis showed that exosomes promoted the M2-type polarization of macrophages, and exosomes overexpressing circSAFB2 further promoted the M2-type polarization of macrophages (**Figure S1**). Taken together, our findings indicated that exosomal circSAFB2 induced M2 macrophage polarization through the miR-620/JAK1/STAT3 axis.

### RCC-Derived Exosomal Circsafb2 Facilitated the Progression of RCC *in Vitro* and *in Vivo* by Inducing Polarization of M2 Macrophages

**Figure 6:**
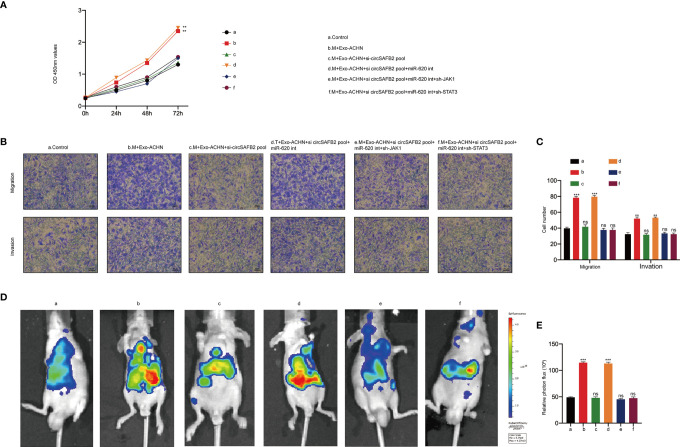
RCC-derived exosomal circSAFB2 facilitates the progression of RCC *in vitro* and *in vivo* by inducing macrophage M2 polarization. **(A)** The proliferation capacity of ACHN cells co-cultured with macrophages subjected to the indicated treatment determined by the CCK8 assay at 0, 24, 48, and 72 hours; **(B, C)** Migration and invasion capacity of Huh7 cells co-cultured with macrophages subjected to the indicated treatment detected by Transwell; **(D, E)** Bioluminescence imaging of mice after ACHN cell injection into the tail vein alone or ACHN cells co-injected with macrophages subjected to the indicated treatment (n=6 per group). Metastasis was monitored and imaged by bioluminescence using an *in-vivo* imaging system. All data are indicated as the mean ± SD; n=3, **P < 0.01, ***P < 0.001. ns, no significance.

To further assess the function of RCC-derived exosomal circSAFB2 in stimulating the progression of RCC, we carried out *in vitro* and *in vivo* experiments. We first analysed the RCC cells proliferation upon co-culture with Exo-ACHN-treated macrophages. The proliferation abilities of ACHN co-cultured with macrophages treated with Exo-ACHN were significantly improved, and miR-620 inhibitor can rescue the tumor-promoting effect lost by circSAFB2 knockdown (**Figure 6A**). We then observed the effects of indirect co-culture of RCC cells and conditioned macrophages by performing *in vitro* Transwell experiments. The migration and invasion abilities of ACHN co-cultured with macrophages treated with Exo-ACHN were significantly improved, while an opposite effect occurred in the circSAFB2-knockdown exosomes (**Figures 6B, C**). Macrophages treated with circSAFB2-knockdown exosomes and miR-620 inhibitor markedly promoted the migration and invasion of ACHN cells (**Figures 6B, C**). Macrophages treated with circSAFB2-knockdown exosomes and miR-620 inhibitor after knockdown of JAK1 or STAT3 in macrophages, indicating that the migration and invasion abilities of JAK1 were significantly reduced (**Figures 6B, C**). Furthermore, we carried out *in vivo* experiments by injecting ACHN cells mixed with conditioned macrophages into mice through the tail vein. After 6 weeks, we observed that ACHN cells mixed with macrophages treated with Exo-ACHN showed significant metastasis compared to the control group (**Figures 6D, E**). However, ACHN cells co-cultured with macrophages treated with circSAFB2-knockdown exosomes showed a lower metastatic ability (**Figures 6D, E**). ACHN cells mixed with macrophages treated with circSAFB2-knockdown exosomes and miR-620 inhibitor underwent significant metastasis *in vivo*. In contrast, further knockdown of JAK1 or STAT3 in macrophages did not increase the *in vivo* metastasis of ACHN cells (**Figures 6D, E**). These *in vitro* and *in vivo* experiments jointly indicated that RCC-derived exosomal circSAFB2 facilitated RCC progression by inducing M2 macrophage polarization.

## Discussion

**Figure 7:**
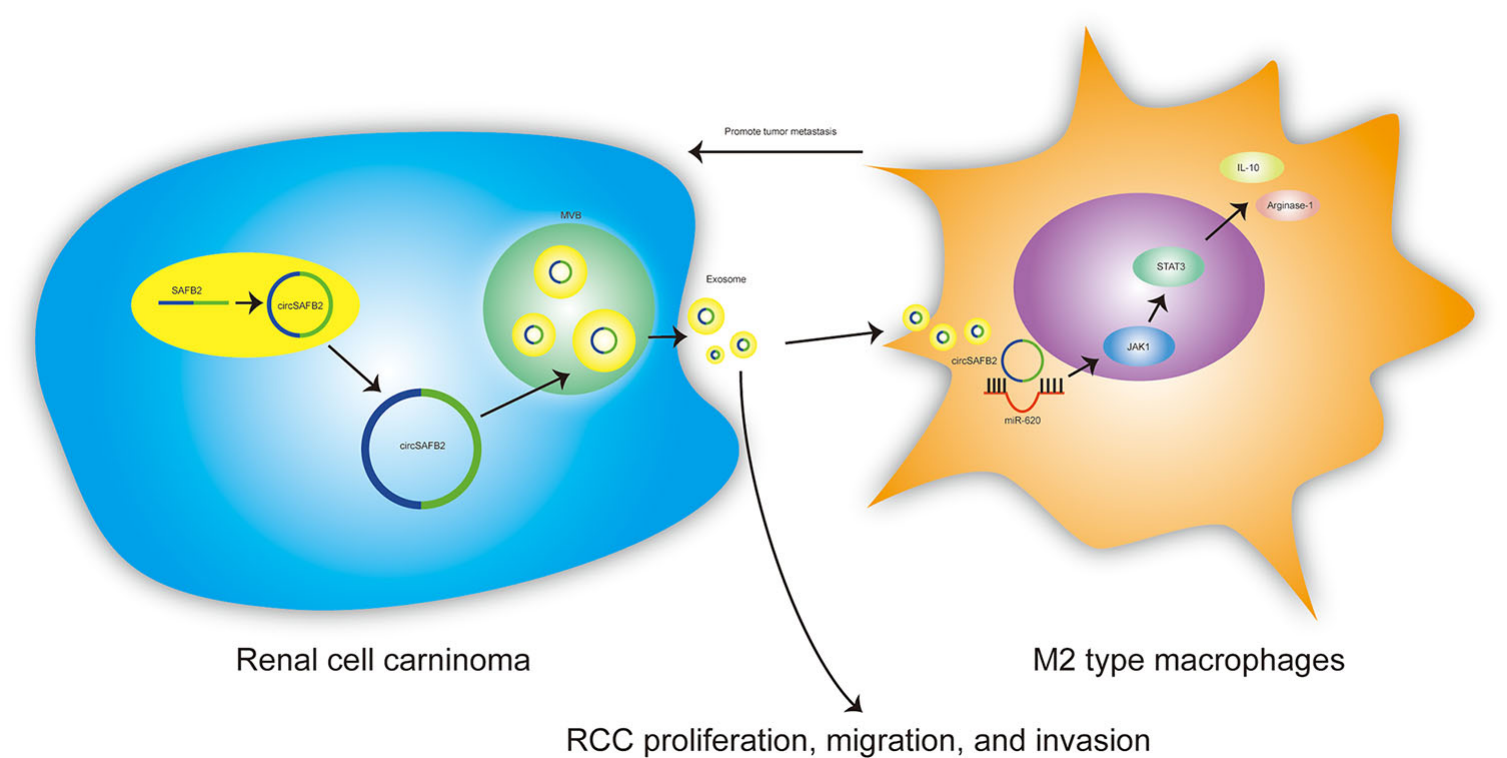
Schematic of the action of tumor-derived exosomal circSAFB2 in mediating M2 macrophage polarization through the miR-620/JAK1/STAT3 axis to promote renal cell carcinoma metastasis.

Exosomes are nano-sized extracellular vesicles released by a variety of cells, which contain cellular contents including small non-coding RNAs, proteins and lipids. The bilayer lipid membrane of exosomes protects these contents from degradation, allowing them for intercellular communication (18). A growing number of mechanistic studies have confirmed that abnormal expression of circRNAs in different cancers can play an important role in regulating tumor immune escape, proliferation, migration, and invasion (19, 20). However, the molecular mechanisms of the circ-RNAs involved in cancer remain unclear. Here, we report that circRNAs derived from SAFB2 and evaluate their biological function in promoting immune escape from RCC. We found that circSAF2 is highly expressed in RCC tissues and RCC-derived exosomes. Furthermore, we demonstrated that RCC-derived exosomal circSAFB2 mediates M2 macrophage polarization *via* the miR-620/JAK1/STAT3 axis to promote RCC metastasis (**Figure 7**). CircSAFB2 may represent a potential target for exosome-mediated tumor immune evasion.

Increasing studies have shown that exosomes generally reflect the malignant characteristics of donor cells and transmit oncogenic signals to recipient cells, thus promoting cancer progression (21). Exosomal circRNAs have been reported to be abnormally expressed in the peripheral blood of a variety of cancer patients, including RCC (21–23). Notably, we identified that circSAFB2 is highly expressed in tissues and exosomes derived from RCC. Furthermore, circSAFB2 was significantly up-regulated in macrophages with the addition of RCC-derived exosomes. Our results emphasize the key role of exosomal circGSE1 in promoting RCC metastasis by mediating M2 macrophage polarization. Furthermore, bioinformatics, luciferase reporter assays, and pull-down assays verified that circSAFB2 directly binds and downregulates miR-620 expression. Furthermore, we found that JAK1 was bioinformatically predicted to be a direct common target of miR-620 and further confirmed the interaction between miR-620 and JAK1 by luciferase reporter assays. JAK1 has been shown to play an important role in inducing M2-type macrophage polarization (16) and STAT3 is an important downstream transcription factor of JAK1 (17). Our data showed that RCC-derived exosomal circSAFB2 mediates the polarization of M2 macrophages *via* the miR-620/JAK1/STAT3 axis and circSAFB2-knockdown can abrogate induced effects. However, inhibition of miR-620 can rescue M2 macrophage polarization defects caused by cirSAFB2 deficiency.

## Conclusion

We have demonstrated that RCC-derived exosomal circSAFB2 mediates the polarization of M2 macrophages *via* the miR-620/JAK1/STAT3 axis to promote RCC metastasis. These findings indicate a molecular mechanism of exosomal circSAFB2 in the progression of RCC and highlight this pathway as a potential diagnostic and therapeutic target.

## Data Availability Statement

The original contributions presented in the study are included in the article/**Supplementary Material**. Further inquiries can be directed to the corresponding author.

## Ethics Statement

The studies involving human participants were reviewed and approved by China Medical University. The patients/participants provided their written informed consent to participate in this study. The animal study was reviewed and approved by China Medical University.

## Author Contributions

XH and JW designed and performed experiments, analyzed data and wrote the manuscript. JH and GG performed the experiments and assisted mouse breeding and analysis of data. JG performed bioinformatic analysis. ZZ and MW assisted in analysis of data, ZS and TX designed and supervised research. All authors read and approved the final manuscript.

## Funding

This work was supported by the following funds:

1. Natural Fund Guidance Plan (82070763; 81770766);
2. Liaoning Provincial Natural Fund Guidance Plan (2019-ZD-0426);
3. Special Fund for Clinical Medicine Research of Chinese Medical Association (20010040796);
4. Liaoning Province Key R&D Guidance Project Plan (2020JH2/10300045; 2019JH8/10300020).
5. Shenyang Science and Technology Plan Population and Health Special Project (19-112-4-031).

## Conflict of Interest

The authors declare that the research was conducted in the absence of any commercial or financial relationships that could be construed as a potential conflict of interest.

## Publisher’s Note

All claims expressed in this article are solely those of the authors and do not necessarily represent those of their affiliated organizations, or those of the publisher, the editors and the reviewers. Any product that may be evaluated in this article, or claim that may be made by its manufacturer, is not guaranteed or endorsed by the publisher.

## Supplementary Material

Supplementary Figure 1 Flow cytometry analysis of the effect of exosomal circSAFB2 on macrophages isolated from fresh blood.

## Abbreviations

RCC, Renal cell carcinoma; CircRNAs, Circular RNAs; NcRNAs, Non-coding RNA; TNF-α, tumor necrosis factor-α; TGF-β, sCCK-8, Cell counting Kit-8; qRT-PCR, Quantitative reverse transcription polymerase chain reaction; IHC, Immunohistochemistry; FISH, Fluorescence *in situ* hybridization; ELISA, Enzyme-linked immunosorbent assay; NTA, Nanoparticle tracking analysis.
