# Supplementary figures and images for: Exosomal Circsafb2 Reshaping Tumor Environment to Promote Renal Cell Carcinoma Progression by Mediating M2 Macrophage Polarization
Source: Front Oncol. 2022 May 12;12:808888. doi: 10.3389/fonc.2022.808888 (PMC9133324; doi:10.3389/fonc.2022.808888)

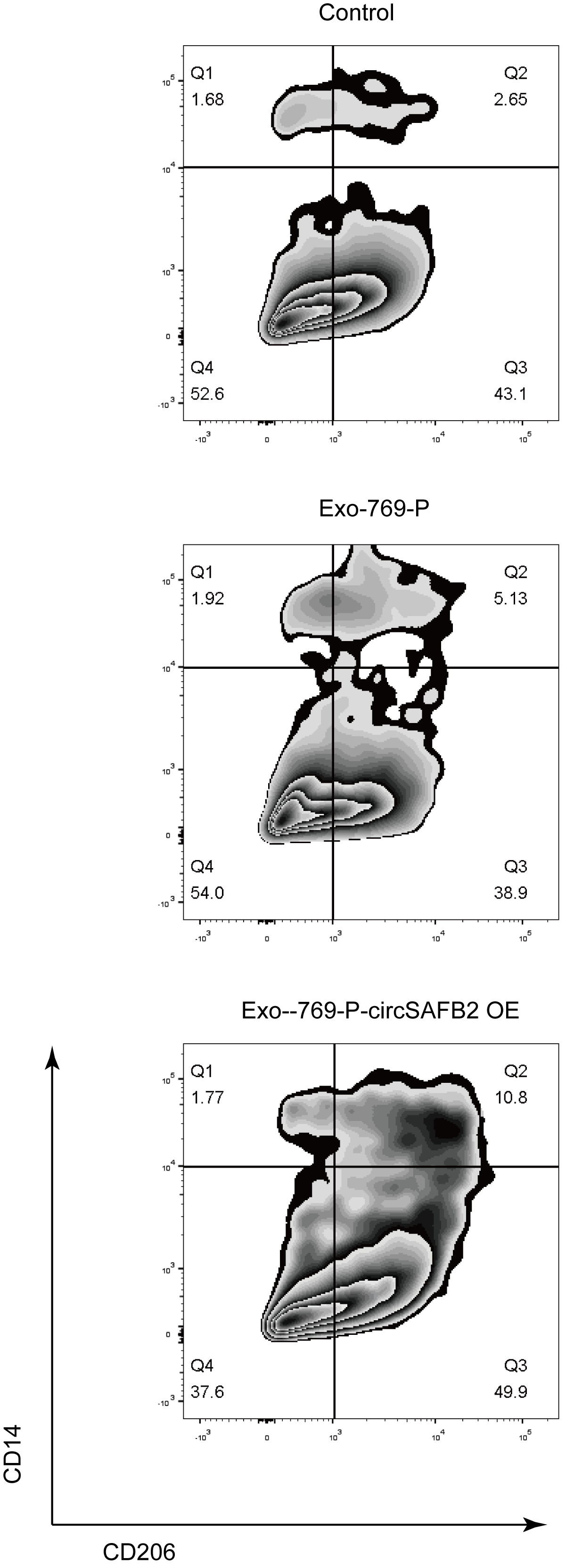

Supplement: Supplementary Figure 1 — Flow cytometry analysis of the effect of exosomal circSAFB2 on macrophages isolated from fresh blood. [file Image_1.tif]
